# The Effects of Nutrient Dynamics on Root Patch Choice

**DOI:** 10.1371/journal.pone.0010824

**Published:** 2010-05-26

**Authors:** Hagai Shemesh, Adi Arbiv, Mordechai Gersani, Ofer Ovadia, Ariel Novoplansky

**Affiliations:** 1 Life Sciences Department, Ben-Gurion University of the Negev, Beer-Sheva, Israel; 2 Miterani Department of Desert Ecology, Blaustein Institutes for Desert Research, Ben-Gurion University of the Negev, Sede Boqer, Israel; University of Copenhagen, Denmark

## Abstract

Plants have been recognized to be capable of allocating more roots to rich patches in the soil. We tested the hypothesis that in addition to their sensitivity to absolute differences in nutrient availability, plants are also responsive to temporal changes in nutrient availability. Different roots of the same *Pisum sativum* plants were subjected to variable homogeneous and heterogeneous temporally – dynamic and static nutrient regimes. When given a choice, plants not only developed greater root biomasses in richer patches; they discriminately allocated more resources to roots that developed in patches with increasing nutrient levels, even when their other roots developed in richer patches. These results suggest that plants are able to perceive and respond to dynamic environmental changes. This ability might enable plants to increase their performance by responding to both current and anticipated resource availabilities in their immediate proximity.

## Introduction

Few, if any, environmental factors remain constant throughout the life of an individual plant [1]. Limited in their ability to relocate, plants have acquired a plethora of deterministic and plastic adaptations that enable them to survive under variable conditions [2], [3], [4], [5]. Due to multiple biotic and abiotic factors, soil nutrients are highly heterogeneous at variable scales [6], [7]. Plastic allocation and positioning of roots enable plants to forage effectively within their immediate environment [8]. Root allocation might vary at two different levels. At the level of the entire plant, resource deficiency usually encourages increased root allocation [9], [10]. However, at the level of individual roots, plants tend to increase their allocation to roots situated in richer patches [1], [11], [12] although such responses vary across species and competitive situations [7]. Such differential root growth exemplifies root foraging, whereby the growth of every individual root depends not only on the absolute quality of its own patch but, through an integrated response of the entire plant, the relative quality of its patch compared to those of other roots of the same plant.

In addition to absolute differences, the dynamics of soil nutrients are likely to vary in both time and space as a result of changes in the rates of organic matter and mineral inputs, decomposition, leaching and competition intensities, which are amplified in water-limited systems such as arctic tundras [e.g. 13] and drylands [e.g. 14].

However, studies of plastic responses to environmental heterogeneity almost invariably conceptualize the environment in terms of discrete static patches or periods [e.g. 15].

The ability to sense not only current absolute values but also the dynamics of environmental processes has been shown in a variety of organisms. For example, parasitic mites move up thermal gradients when foraging for hosts [16], and birds navigate up gradients of indicatory volatile organic compounds [17]. Perhaps the most studied example of gradient perception is chemotaxis in certain motile bacteria, which are capable of perceiving and adaptively responding to the spatial attributes of resource gradients, registering nutrient concentrations at different points in time [e.g. 18], [19]. The existence of such abilities in rudimentary life forms such as bacteria suggests that the perception of environmental gradients might also occur in additional organisms which lack central nervous systems.

Recent studies have demonstrated that plants are able to respond to temporally and spatially dynamic changes in resource availabilities [for a review see 20]. For example, *Trifolium repens* plants which developed in vertically increasing spatial light gradients grew longer petioles than controls, which experienced constant high or low light [21]. Similarly, *Calendula arvensis* and *Phlox glandiflora* grown in dynamically-increasing rooting volumes developed larger and had greater fitness compared to plants that were grown in the largest, yet constant, rooting volume [22].

Here, we tested the hypothesis that plants are able to perceive and respond to temporal changes in resource availability at the scale of the single root system. Specifically, we predicted that, regardless of absolute resource availability, plants would preferentially allocate more resources to roots experiencing increasing nutrient availabilities and would discriminate against roots experiencing decreasing nutrient levels. To test these hypotheses, we studied the responsiveness and performance of split-root plants whose roots were subjected to variable, temporally dynamic and static nutrient regimes.

## Materials and Methods

*Pisum sativum* L. cv Kelvedon Wonder was used due to the relative ease of its rearing and manipulation and its Mediterranean origin, which is characterized by substantial temporal and spatial variabilities in resource availability at the fine scales of individual plants and organs [e.g. 23], [24]. Young ‘split-root’ seedlings were grown following Gersani & Sachs (1992), so that they developed two equal roots following removal of the tip of the seminal root. Seedlings were planted so each of their roots was grown in a separate 400 ml drainable plastic pot filled with grade-3 vermiculite. Nutrient solutions were prepared using a 20-20-20 NPK fertilizer with microelements (Poly-Feed; Haifa Chemicals, Haifa, Israel).

Each of the two individual roots was provided with one of the following nutrient solutions: high (HIGH; 0.225 g L^−1^), average (AVE; 0.125 g L^−1^), low (LOW; 0.025 g L^−1^), increasing (INC; from 0.025 to 0.225 g L^−1^) and decreasing (DEC; from 0.225 to 0.025 g L^−1^). The long-term average nutrient level was the same in the INC, DEC and AVG regimes. The experimental design included three stationary (LOW, AVE & HIGH) and two dynamic regimes (INC & DEC), whose pair-wise combinations included a total of 15 treatments, 24 replications per treatment and a total of 360 plants. Nutrient levels used in our experiment fall below the range found optimal in a previous experiment under similar conditions [11].

Each pot received 100 ml of nutrient solution (200 ml per plant) twice a week. In order to prevent nutrient accumulation, all pots were flushed weekly by tap water prior to nutrient supplementation. Treatments were initiated one day after planting. In the dynamic regimes (INC, DEC), the concentrations of the nutrient solutions were changed weekly following linear trajectories.

The experiment was conducted in a glasshouse at Ben-Gurion University, Beer-Sheva, Israel (31°14′ N, 34°48′ E), under natural light (70% of ambient). Plants were assigned to blocks according to their initial leaf number. Each block contained 15 plants with a total of 24 blocks. Ten blocks were harvested after 4.5 weeks, the rest were harvested 9 weeks into the experiment.

At harvest, the two root systems of each plant were separated; scanned using a root scanner (EPSON LA 2400) and their length was estimated using the Win-Rhizo software (Regent Instruments, Canada). Plant biomass was estimated using an analytical scale (Sartorius, Germany) after drying at 60° C for 72 h.

Root data is only represented by dry root biomass, because throughout, root morphological attributes were tightly correlated with root biomass (data not shown) and no differences in the correlations could be found between the treatments (Table S1). Data regarding total plant, shoot, root and reproductive masses as a function of treatment as well as additional technical information is available in the electronic supplementary material (Fig. S1, S2, S3, S4, S5, Text S1).

Root biomass was compared at two different levels: a) treatment differences, in total mass of both root systems, b) regime differences, between the two roots of the same plant. The comparisons were made among and within each of the following treatment groups:

1. Homogeneous treatments, where both roots experienced identical regimes: INC-INC, DEC-DEC, LOW-LOW, AVE-AVE and HIGH-HIGH.
2. Heterogeneous stationary treatments, where the roots experienced different stationary regimes: HIGH-AVE, HIGH-LOW and AVE-LOW.
3. Heterogeneous treatments including the INC regime: INC-DEC, INC-LOW, INC-AVE and INC-HIGH.
4. Heterogeneous treatments including the DEC regime: DEC-LOW, DEC-AVE and DEC-HIGH, excluding the INC-DEC treatment, which was included in group 3.

The first and second treatment groups tested the plants' responsiveness to constant differences in nutrient availability at the levels of entire plant and single roots, respectively. The third and fourth treatment groups tested the plants' responsiveness to dynamically- increasing and decreasing nutrient levels, respectively.

Because the two root systems of each plant were interdependent, their performances were analyzed using split plot ANOVAs, with the two root systems acting as the within-subject factor and the nutrient treatment as the between-subject factor. Accordingly, a significant regime (within subject factor) effect indicated an overall difference between the two parts of paired root systems, regardless of treatment. A significant treatment (between subject factor) effect indicated an overall difference, at the entire plant level, between plants experiencing different treatments. A significant regime by treatment interaction indicated that differences between the two parts experiencing different nutrient regimes varied significantly between treatments. In order to verify that the regime effects were consistent among plants within each treatment, we conducted Wilcoxon Signed Rank Tests [25]. This none-parametric test allowed us to rule out the possibility that the observed regime differences were obtained by chance, due to a small number of observations in which this pattern was evident. Variables characterising the entire plant such as total, shoot, vegetative and reproductive (flowers + pods) biomasses as well as root allocation were analyzed using one-way ANOVAs, with treatment as an independent factor. ANOVAs were followed by post-hoc Tukey comparisons. None of the plants flowered by the time of the interim harvest, reproductive biomass was therefore analyzed only for the final harvest.

## Results

### Whole-plant attributes

**Figure 1:**
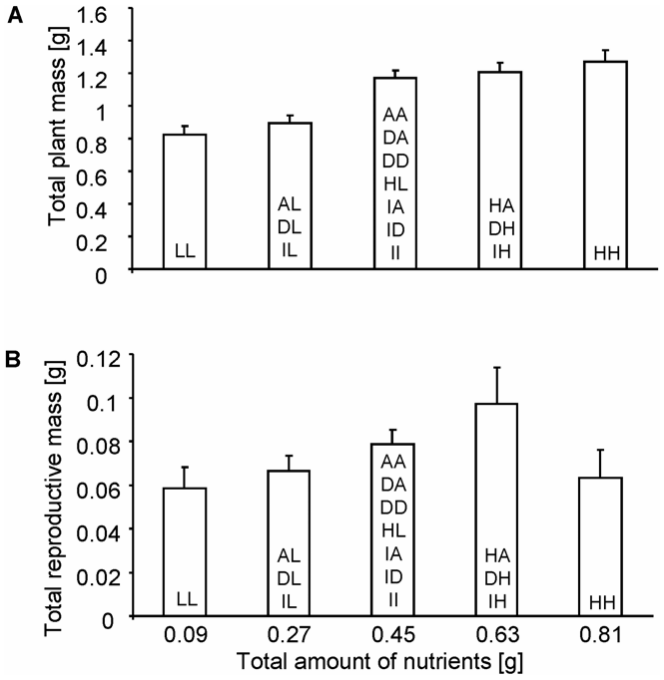
The effect of total nutrient availability on total and reproductive mass. Total plant (a) and reproductive (b) biomass as a function of the total amount of nutrients each plant received throughout the entire experiment. Nutrients were supplied as 20-20-20 fertilizer (for more data see the Materials and Methods). Data presented are for the final harvest. Treatments contributing to each bar are indicated within it. Nutrient regimes: INC (I), DEC (D), HIGH (H), AVE (A), LOW (L). Values are means ±1 S.E.

The total amount of nutrients applied to the entire plant over the whole experimental period, (total mass of fertilizer given to the plant) did not affect total plant biomass by the interim harvest (F_12,148_ = 1.413, P = 0.166). However, by the final harvest, plant biomass was positively correlated with nutrient availability (F_4,197_ = 7.843, P<0.001, Fig. 1a). These differences translated into treatment differences, with total plant, vegetative and total root biomasses being 54, 62 and 44% greater in HIGH-HIGH than in LOW-LOW, respectively (Fig. S1, S2, S3).

In both harvests, treatment had inconsistent affects on root allocation (Fig. S4, Table S2). Experiment-long nutrient availability had no significant effects on the total reproductive biomass (F_4,197_ = 1.525, P = 0.196, Fig. 1b). In addition, treatment had no consistent effect on total reproductive biomass, although INC-HIGH and AVE-AVE had non-significantly higher values than other treatments (Fig. S5, Table S2).

### Biomass allocation to individual root systems

**Figure 2:**
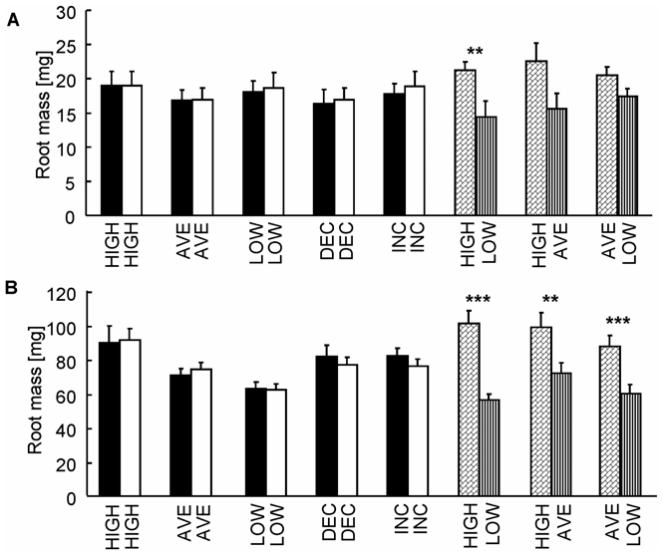
Patch choice in the homogenous and stationary heterogeneous treatments. Biomass of individual roots of split-root plants at the interim (a) and final (b) harvests of homogenous (black and white bars) and stationary heterogeneous (striped and checkered bars) treatments. Pair-wise comparisons were done using Wilcoxon Signed-rank Test; * <0.05, ** <0.01, *** <0.001. Values are means ±1 S.E.

**Figure 3:**
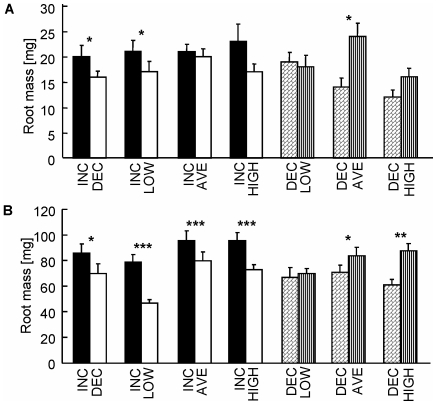
Patch choice in the increasing and decreasing treatments. Biomass of individual roots of split-root plants at the interim (a) and final (b) harvests of treatments that included dynamic nutrient regimes. Treatments including the INC regime are depicted by black and white bars, and treatments including the DEC regime are depicted by striped and checkered bars. Pair-wise comparisons were done using Wilcoxon Signed-rank Test; * <0.05, ** <0.01, *** <0.001. Values are means ±1 S.E.

As expected, in both harvests there was a significant group by regime interaction (F_3,156_ = 9.052, P<0.001 and F_3,185_ = 45.478, P<0.001 for the 1^st^ & 2^nd^ harvests, respectively; Figs. 2,3), indicating that the patterns of root allocation within plants differed between the four treatment groups (Homogeneous, Heterogeneous stationary, Heterogeneous including the INC, Heterogeneous including the DEC. For more details see Methods). Indeed, in neither harvest were significant differences found between the biomass of the two individual roots of the same plant that developed under the same regimes (homogenous group, Fig. 2, Table 1). Plants experiencing two different static regimes developed greater root biomass under higher nutrient availabilities (stationary heterogeneous group, Fig. 2, Table 1). In both harvests, root biomass was significantly greater in INC than in all other regimes, except for INC-AVE and INC-HIGH at the interim harvest (Fig. 3, Table 1). For example, by the middle of the experiment, INC-DEC plants had 27% greater root biomass in INC than in DEC, in spite of a 133% greater average nutrient availability in DEC than in INC during that period (Fig. 3a, Table 1). By the final harvest, root biomass under DEC was indistinguishable (P>0.05) from LOW and significantly lower by 16 and 30% than AVE and HIGH, respectively. This was true in spite of the identical cumulative nutrient availability experienced by roots grown under DEC and AVE regimes, and the four-fold greater nutrient availability experienced by the DEC compared to LOW (Fig. 3b).

**Table 1:** Statistical analysis.

		Interim harvest			Final harvest		
		Df	F	P	Df	F	P
**Homogenous treatments**	Block	11,36	0.893	0.556	12,46	1.777	0.081
	Regime	1,36	0.545	0.465	1,46	0.965	0.331
	Treatment	4,36	0.410	0.800	4,46	4.547	**0.003**
	Reg.×Treat	4,36	0.046	0.996	4,46	0.487	0.745
**Heterogeneous treatments**	Block	10,18	1.401	0.256	12,21	1.464	0.214
	Regime	1,18	14.315	**0.001**	1,21	67.674	**<0.001**
	Treatment	2,18	0.121	0.887	2,21	0.223	0.801
	Reg.×Treat	2,18	0.683	0.518	2,21	1.980	0.163
**Increasing treatments**	Block	10,31	0.830	0.604	12,35	1.912	0.067
	Regime	1,31	5.181	**0.030**	1,35	61.705	**<0.001**
	Treatment	3,31	0.250	0.861	3,35	8.847	**0.002**
	Reg.×Treat	3,31	0.576	0.635	3,35	1.457	0.243
**Decreasing treatments**	Block	10,20	0.657	0.749	12,25	1.934	0.079
	Regime	1,20	4.238	0.053	1,25	14.188	**<0.001**
	Treatment	2,20	5.069	**0.017**	2,25	0.811	0.455
	Reg.×Treat	2,20	4.388	**0.026**	2,25	4.722	**0.018**

The effects of the experimental treatments (whole plant level) and nutrient regimes (single root system level) on root biomass. Results presented are of a split-plot ANOVA with Regime as the within subject factor and Treatment as the between subject factor. Different error terms were used for the between and within comparisons.

## Discussion

Plants are able to discriminately allocate greater resources to individual organs that grow under preferable conditions [Fig. 2; 7,26], and at the time – even at the expense of other organs on the same plant [11], [27], [28]. However, our results demonstrate that root development might also be responsive to changes in nutrient supply, regardless of, and at times even in clear contrast to, the pattern expected based on absolute nutrient availability: when given a choice, individual plants almost invariably allocated more resources to roots growing under increasing nutrient supply, even when their other root was growing under higher resource availability (INC-HIGH; Fig. 3b). Similarly, when both roots grew under the same cumulative nutrient supply (DEC-AVE), plants developed greater root biomass under stationary average supply than under decreasing nutrient supply (Fig. 3). These findings, and similar results obtained from additional experiments with *Portulaca oleracea* (Portulacaceae) and *Emex spinosa* (Polygonaceae) (Arbiv & Shemesh, unpublished data), suggest that plants develop their roots, not only according to absolute nutrient levels, but also according to changes in nutrient availability.

A seeming alternative interpretation to our results is that optimal resource supply increases with plant size, whereas constant resource supply might exceed the plant's demand and interfere with its performance at a young age. Indeed, according to the “steady-state nutrition” approach used in agriculture and forestry, plants perform better if provided with increasing nutrient levels which prevent build-up of super-optimal nutrient concentrations in the potting medium due to slow uptake at a young age [29]. However, in our experiment, nutrient levels were kept constant and below harmful levels by frequent flushing and replenishing of the nutrient solutions. This was evident by the invariably greater resource allocation to roots growing under HIGH regime in the stationary treatments (Fig. 2). Furthermore, in plants with a short growing season, early luxury consumption is selected for by temporal variability in nutrient availability [30] and strong competition for nutrients [31]. This implies that high nutrient availability early in the season is not only harmless, but might also be favourable to annual plants such as those experimented with in the current study.

Interestingly, the results demonstrated significant effects of nutrient dynamics on root discrimination, but only minor effects on total plant growth and reproduction (Fig. S1b). These findings might be attributed to the plants' ability to integrate over environmental heterogeneity by plastically adjusting their overall root allocation [10], shoot size [9] and physiological attributes [for reviews see 32], [33]. Accordingly, further studying of root responsiveness to resource gradients should include experiments where whole plants are grown for longer periods and individual roots are subjected to even wider ranges of relative and absolute nutrient levels.

Responsiveness to resource gradients requires the integration of environmental information [34]. Plants are able to compare growth directions and patches and discriminate between them according to their relative adaptive values by employing various tropic and nastic movements [35], [e.g. 36], [37] and discriminatory development [1], [26], [e.g. 38], [39]. Interestingly, plants are also able to integrate environmental stimuli and experiences over time [40]. Examples of “plant memory” include the abilities to perceive and adaptively respond to weather changes [[vernalization; e.g. 41], past mechanical stimulation [e.g. 42], damage [e.g. 43] and stresses [priming]; [ 44,45]. As most environmental variables significantly vary in both space and time, their stationary levels often have only limited predictive values [46]. Therefore, the ability to perceive not only stationary levels but also the dynamics of environmental factors might assist organisms to navigate along spatial gradients [e.g. 17], [e.g. 18] and anticipate future conditions by perceiving temporal changes [e.g. 47]. Our results suggest that plants too, are responsive to environmental trajectories which might enable them to anticipate future resource distributions and affectively forage for plentiful opportunities in time [20]. Because the tips of growing roots simultaneously experience both spatial and temporal gradients, the greater sensitivity of the root apex to environmental stimuli [36], [48], suggests that the perception of temporal and spatial gradients is based on the same mechanisms, however, testing this hypothesis requires additional experimentation.

Environmental variability and its implications for survival, distribution, movement and life-history attributes are pivotal in understanding organismic ecology and evolution. However, until recently, environmental change per se, i.e. independently of stationary environmental states, has received only limited attention. Beyond the need to further study the ecological implications and underlying mechanisms of gradient responsiveness, our results exemplify the yet to be explored significance of dynamic processes and the potential role for their information content in ecological systems.

## Supporting Information

Text S1 Technical information not mentioned in the Materials and Methods.
(0.03 MB DOC)

Table S1 Correlations between root length, volume, and biomass.
(0.03 MB DOC)

Table S2 One-way ANOVAs for the effects of treatment on plant performance at the interim and final harvests.
(0.03 MB DOC)

Figure S1 Total root biomass in the interim (a) and final (b) harvests. Nutrient regimes: INC (I), DEC (D), HIGH (H), AVE (A), LOW (L). Values are means ±1 S.E.
(0.43 MB TIF)

Figure S2 Vegetative shoot biomass in the interim (a) and final (b) harvests. Nutrient regimes: INC (I), DEC (D), HIGH (H), AVE (A), LOW (L). Letters indicate significant differences (Tukey), Bars lacking letters do not differ from any other bar. Values are means ±1 S.E.
(0.44 MB TIF)

Figure S3 Total plant biomass in the interim (a) and final (b) harvests. Nutrient regimes: INC (I), DEC (D), HIGH (H), AVE (A), LOW (L). Letters indicate significant differences (Tukey), Bars lacking letters do not differ from any other bar. Values are means ±1 S.E.
(0.43 MB TIF)

Figure S4 Root allocation in the interim (a) and final (b) harvests. Nutrient regimes: INC (I), DEC (D), HIGH (H), AVE (A), LOW (L). Values are means ±1 S.E.
(0.43 MB TIF)

Figure S5 Reproductive mass in the final harvest. Nutrient regimes: INC (I), DEC (D), HIGH (H), AVE (A), LOW (L). Values are means ±1 S.E.
(0.38 MB TIF)
