# Supplementary material for: The Effects of Nutrient Dynamics on Root Patch Choice
Source: PLoS One. 2010 May 26;5(5):e10824. doi: 10.1371/journal.pone.0010824 (PMC2877079; doi:10.1371/journal.pone.0010824)
Supplement: Text S1 — Technical information not mentioned in the Materials and Methods. (0.03 MB DOC) [file pone.0010824.s001.doc]

**Technical information (not mentioned in the Materials and Methods)**

**Plant materials and growth conditions:** We chose to use cultivated legumes due to the relative ease of their manipulation and readiness of their responses to nutrient heterogeneity (Gersani et al. 1998). All seeds were germinated in vermiculite. Three days from germination, the seminal root was severed two mm below the hypocotyl and the plants were replanted in damp vermiculite. Seven days from germination, the stump of the seminal root typically regenerated three lateral roots that were thinned down to two roots. The seedlings were positioned so their stems were located above the adjacent rims of the two pots.

**Experimental design:** Each block contained one replicate of each treatment, resulting in 15 plants per block with a total of 24 blocks. Plants were spaced 25 cm apart to minimize light competition. The experiment started on December 17, 2007, which fits the natural growth season of peas.
