# Supplementary material for: The Effects of Nutrient Dynamics on Root Patch Choice
Source: PLoS One. 2010 May 26;5(5):e10824. doi: 10.1371/journal.pone.0010824 (PMC2877079; doi:10.1371/journal.pone.0010824)
Supplement: Table S1 — Correlations between root length, volume, and biomass. (0.03 MB DOC) [file pone.0010824.s002.doc]

|  | Root 1 | | Root 2 | |
| --- | --- | --- | --- | --- |
| **Variables** | Mass – Length | Mass - Volume | Mass – Length | Mass - Volume |
| **R** | 0.73 | 0.74 | 0.73 | 0.72 |
| **Bonferroni Probabilities** | P<0.001 | P<0.001 | P<0.001 | P<0.001 |
| **Treatment X variable** | 0.247 | 0.288 | 0.684 | 0.503 |
