# Supplementary material for: The Effects of Nutrient Dynamics on Root Patch Choice
Source: PLoS One. 2010 May 26;5(5):e10824. doi: 10.1371/journal.pone.0010824 (PMC2877079; doi:10.1371/journal.pone.0010824)
Supplement: Table S2 — One-way ANOVAs for the effects of treatment on plant performance at the interim and final harvests. (0.03 MB DOC) [file pone.0010824.s003.doc]

|  | Interim harvest | | | Final harvest | | |
| --- | --- | --- | --- | --- | --- | --- |
|  | Df | F | P | Df | F | P |
| Total plant mass | 14,146 | 1.236 | 0.255 | 14,187 | 2.890 | <0.001 |
| Vegetative shoot mass | 14,146 | 1.170 | 0.304 | 14,187 | 3.841 | <0.001 |
| Total root mass | 14,146 | 1.375 | 0.172 | 14,187 | 1.571 | 0.091 |
| Root allocation | 14,146 | 1.696 | 0.062 | 14,187 | 0.593 | 0.869 |
| Reproductive mass | NA | NA | NA | 14,187 | 1.093 | 0.366 |
