# Supplementary figures and images for: The Effects of Nutrient Dynamics on Root Patch Choice
Source: PLoS One. 2010 May 26;5(5):e10824. doi: 10.1371/journal.pone.0010824 (PMC2877079; doi:10.1371/journal.pone.0010824)

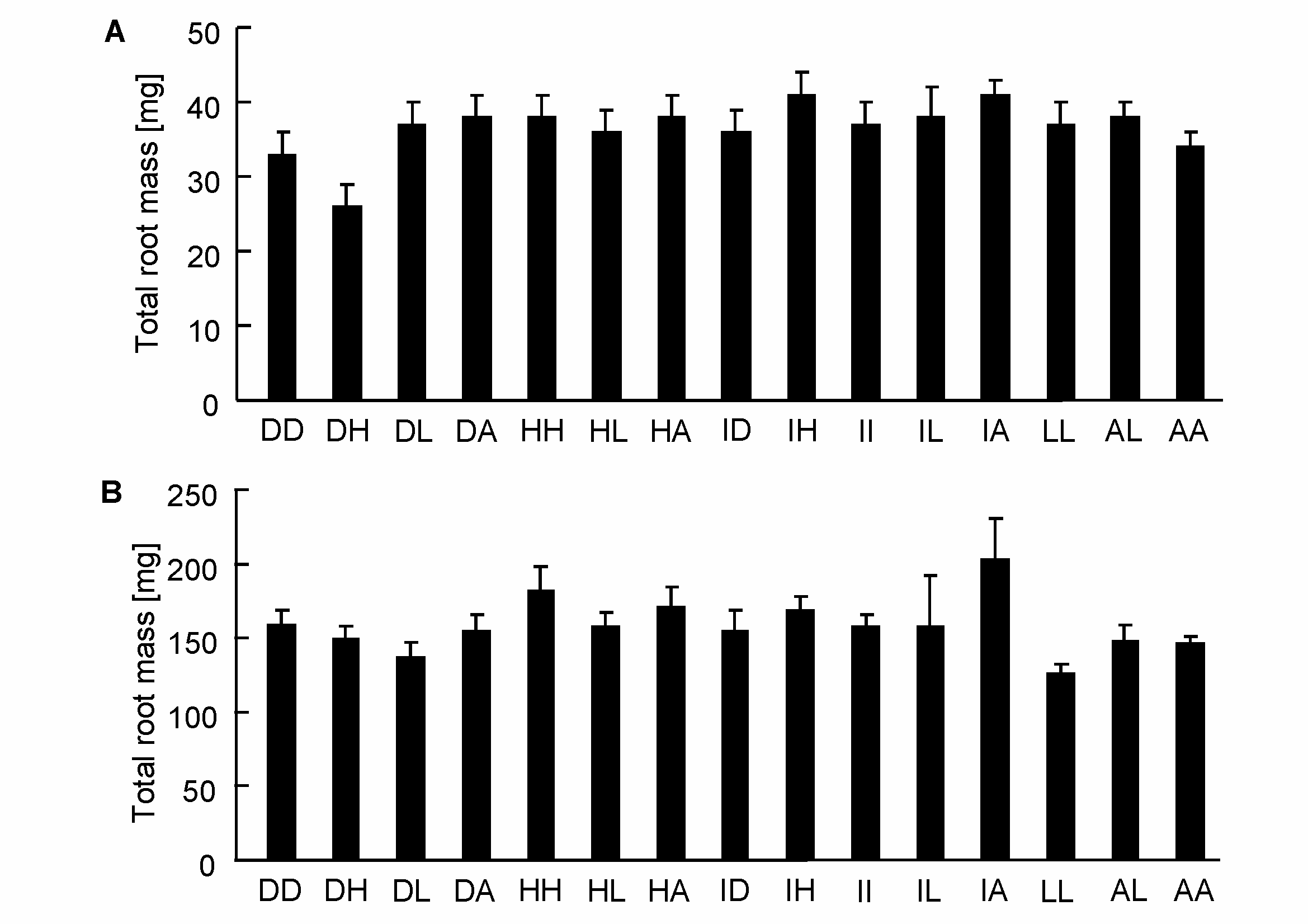

Supplement: Figure S1 — Total root biomass in the interim (a) and final (b) harvests. Nutrient regimes: INC (I), DEC (D), HIGH (H), AVE (A), LOW (L). Values are means ±1 S.E. (0.43 MB TIF) [file pone.0010824.s004.tif]

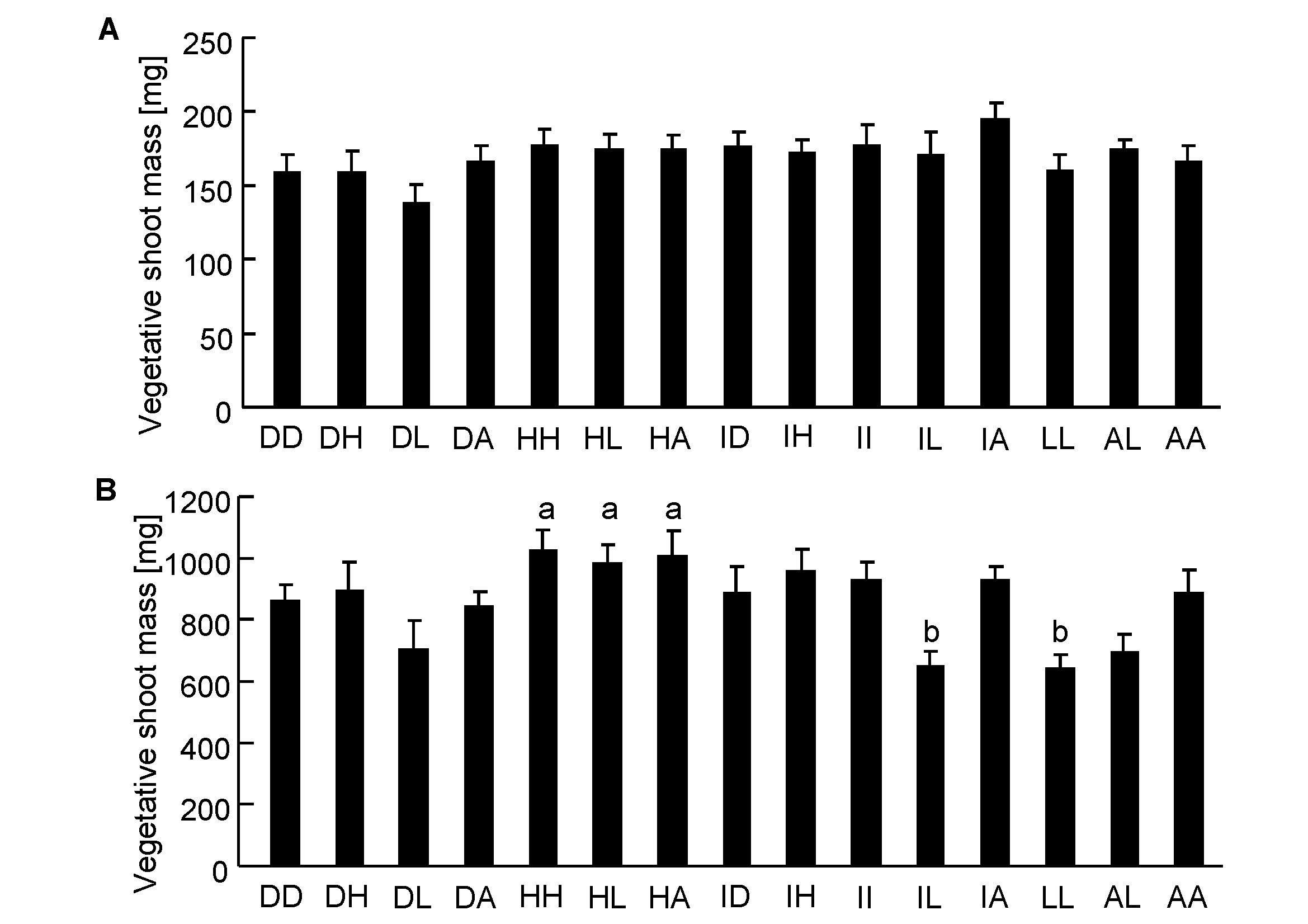

Supplement: Figure S2 — Vegetative shoot biomass in the interim (a) and final (b) harvests. Nutrient regimes: INC (I), DEC (D), HIGH (H), AVE (A), LOW (L). Letters indicate significant differences (Tukey), Bars lacking letters do not differ from any other bar. Values are means ±1 S.E. (0.44 MB TIF) [file pone.0010824.s005.tif]

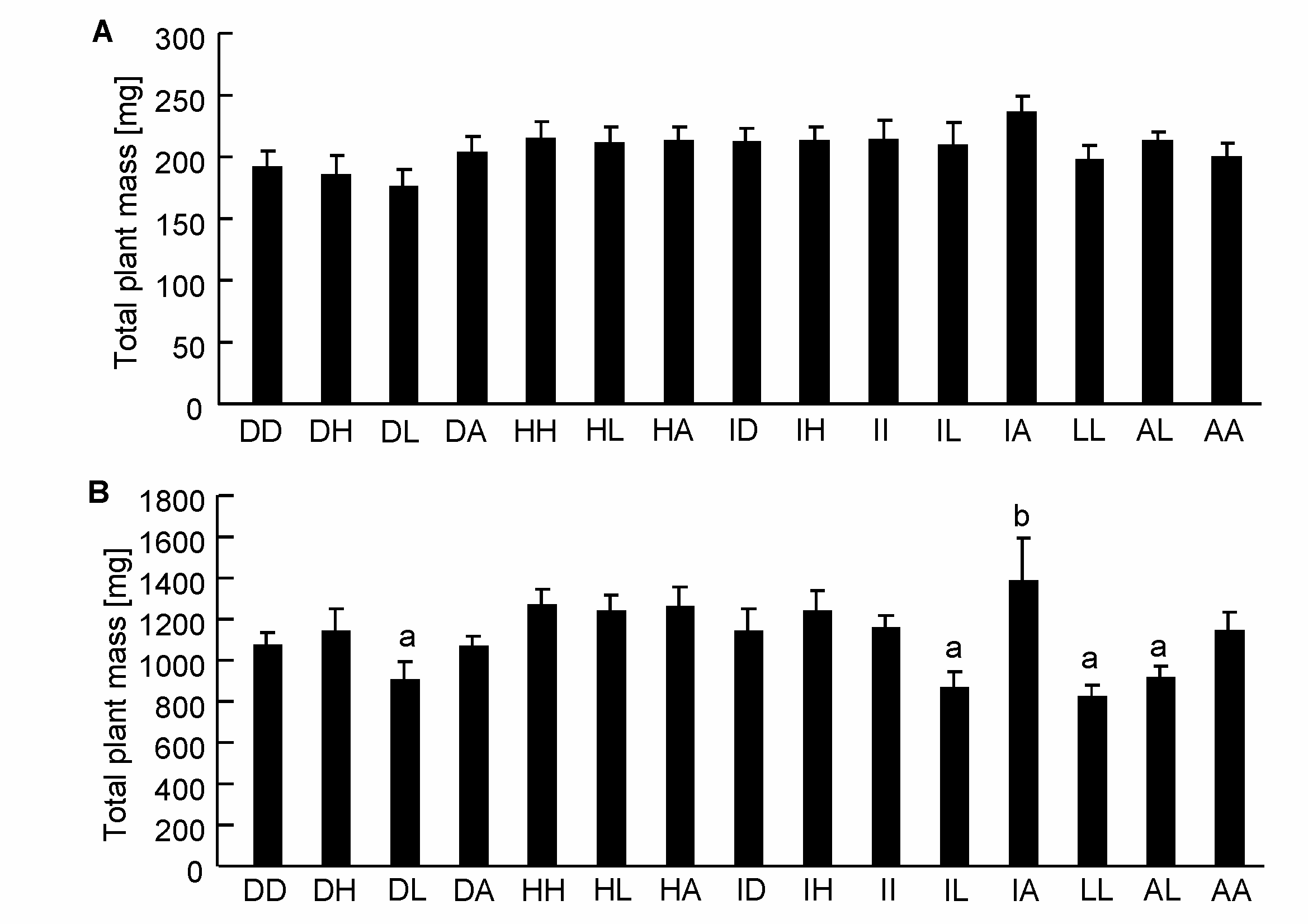

Supplement: Figure S3 — Total plant biomass in the interim (a) and final (b) harvests. Nutrient regimes: INC (I), DEC (D), HIGH (H), AVE (A), LOW (L). Letters indicate significant differences (Tukey), Bars lacking letters do not differ from any other bar. Values are means ±1 S.E. (0.43 MB TIF) [file pone.0010824.s006.tif]

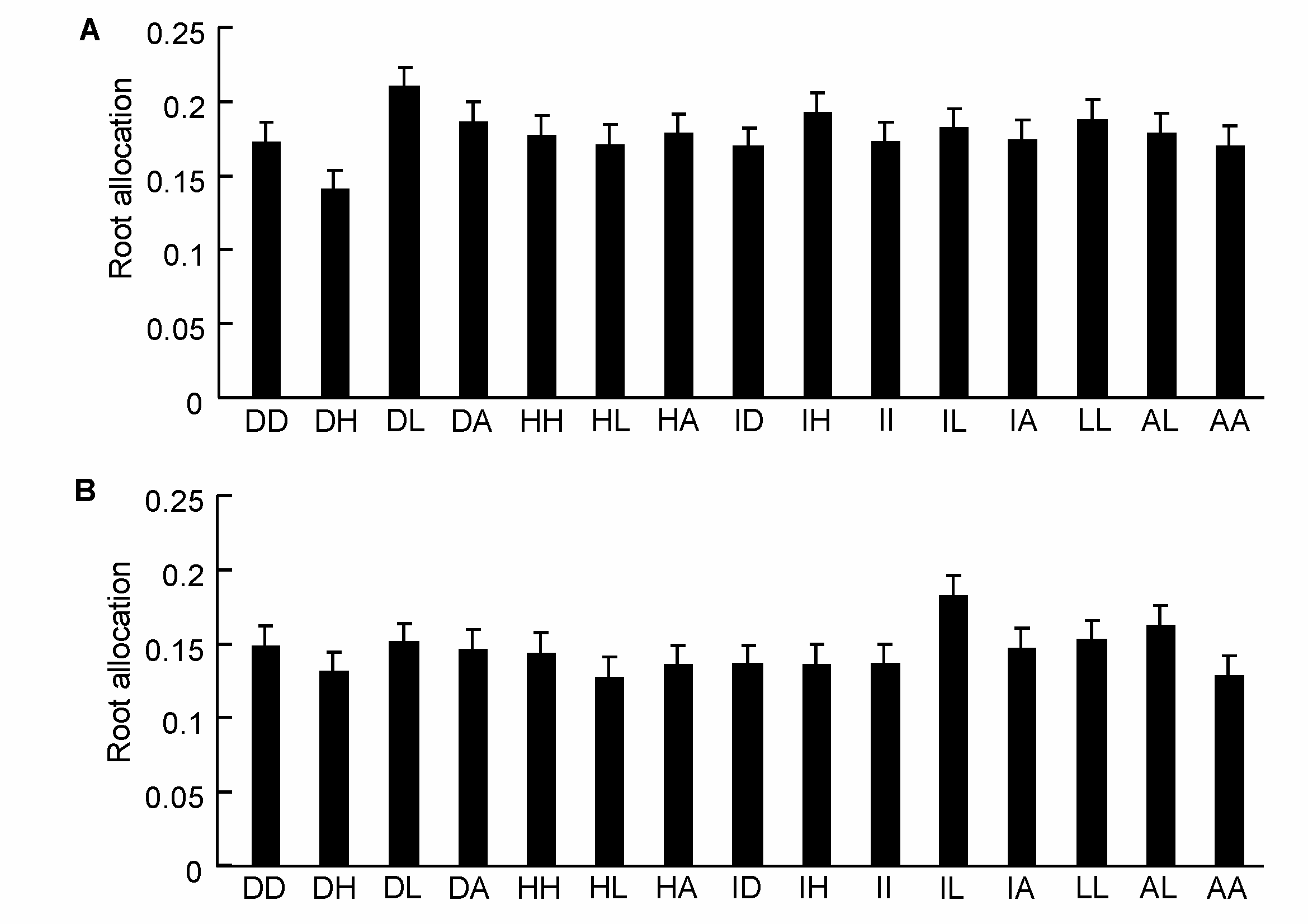

Supplement: Figure S4 — Root allocation in the interim (a) and final (b) harvests. Nutrient regimes: INC (I), DEC (D), HIGH (H), AVE (A), LOW (L). Values are means ±1 S.E. (0.43 MB TIF) [file pone.0010824.s007.tif]

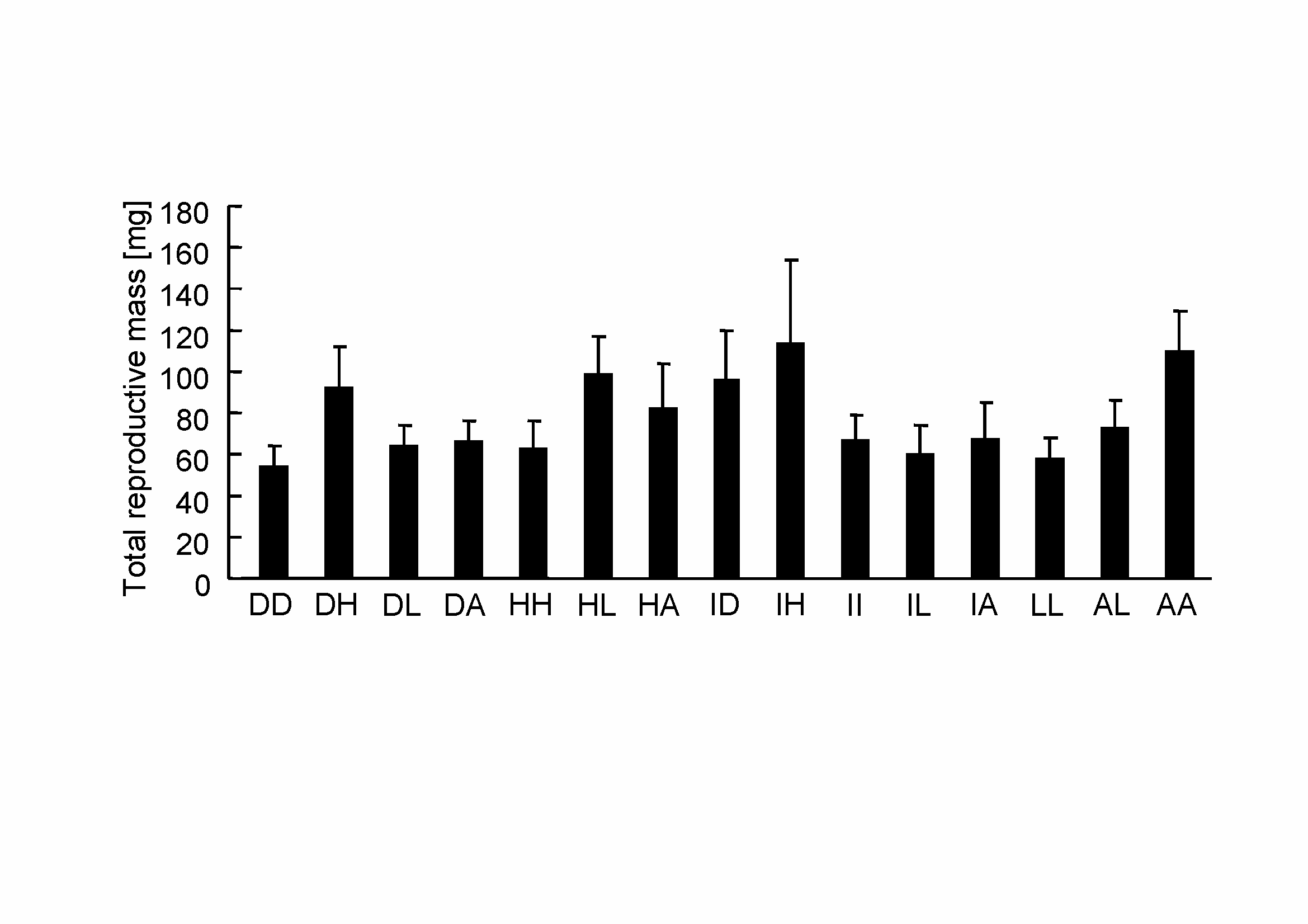

Supplement: Figure S5 — Reproductive mass in the final harvest. Nutrient regimes: INC (I), DEC (D), HIGH (H), AVE (A), LOW (L). Values are means ±1 S.E. (0.38 MB TIF) [file pone.0010824.s008.tif]
